# Factors Associated With Acute Pain Estimation, Postoperative Pain Resolution, Opioid Cessation, and Recovery: Secondary Analysis of a Randomized Clinical Trial

**DOI:** 10.1001/jamanetworkopen.2019.0168

**Published:** 2019-03-01

**Authors:** Jennifer M. Hah, Eric Cramer, Heather Hilmoe, Peter Schmidt, Rebecca McCue, Jodie Trafton, Debra Clay, Yasamin Sharifzadeh, Gabriela Ruchelli, Stuart Goodman, James Huddleston, William J. Maloney, Frederick M. Dirbas, Joseph Shrager, John G. Costouros, Catherine Curtin, Sean C. Mackey, Ian Carroll

**Affiliations:** 1 Division of Pain Medicine, Department of Anesthesiology, Perioperative, and Pain Medicine, Stanford University, Palo Alto, California; 2 Stanford Systems Neuroscience and Pain Lab, Stanford University, Palo Alto, California; 3 Psychiatry and Behavioral Sciences, Stanford University, Palo Alto, California; 4 Veterans Administration Program Evaluation and Resource Center, Veterans Health Administration Office of Mental Health Operations, Menlo Park, California; 5 Department of Orthopaedic Surgery, Stanford University, Redwood City, California; 6 Department of Bioengineering (by courtesy), Stanford University, Redwood City, California; 7 Department of Orthopaedic Surgery, Stanford University, Palo Alto, California; 8 Department of General Surgery, Stanford University, Palo Alto, California; 9 Cardiothoracic Surgery, Division of Thoracic Surgery, Stanford University, Palo Alto, California; 10 Division of Hand and Plastic Surgery, Department of Orthopaedic Surgery, Stanford University, Palo Alto, California

## Abstract

**Importance:**

Acute postoperative pain is associated with the development of persistent postsurgical pain, but it is unclear which aspect is most estimable.

**Objective:**

To identify patient clusters based on acute pain trajectories, preoperative psychosocial characteristics associated with the high-risk cluster, and the best acute pain predictor of remote outcomes.

**Design, Setting, and Participants:**

A secondary analysis of the Stanford Accelerated Recovery Trial randomized, double-blind clinical trial was conducted at a single-center, tertiary, referral teaching hospital. A total of 422 participants scheduled for thoracotomy, video-assisted thoracoscopic surgery, total hip replacement, total knee replacement, mastectomy, breast lumpectomy, hand surgery, carpal tunnel surgery, knee arthroscopy, shoulder arthroplasty, or shoulder arthroscopy were enrolled between May 25, 2010, and July 25, 2014. Data analysis was performed from January 1 to August 1, 2018.

**Interventions:**

Patients were randomized to receive gabapentin (1200 mg, preoperatively, and 600 mg, 3 times a day postoperatively) or active placebo (lorazepam, 0.5 mg preoperatively, inactive placebo postoperatively) for 72 hours.

**Main Outcomes and Measures:**

A modified Brief Pain Inventory prospectively captured 3 surgical site pain outcomes: average pain and worst pain intensity over the past 24 hours, and current pain intensity. Within each category, acute pain trajectories (first 10 postoperative pain scores) were compared using a k-means clustering algorithm. Fifteen descriptors of acute pain were compared as predictors of remote postoperative pain resolution, opioid cessation, and full recovery.

**Results:**

Of the 422 patients enrolled, 371 patients (≤10% missing pain scores) were included in the analysis. Of these, 146 (39.4%) were men; mean (SD) age was 56.67 (11.70) years. Two clusters were identified within each trajectory category. The high pain cluster of the average pain trajectory significantly predicted prolonged pain (hazard ratio [HR], 0.63; 95% CI, 0.50-0.80; *P* < .001) and delayed opioid cessation (HR, 0.52; 95% CI, 0.41-0.67; *P* < .001) but was not a predictor of time to recovery in Cox proportional hazards regression (HR, 0.89; 95% CI, 0.69-1.14; *P* = .89). Preoperative risk factors for categorization to the high average pain cluster included female sex (adjusted relative risk [ARR], 1.36; 95% CI, 1.08-1.70; *P* = .008), elevated preoperative pain (ARR, 1.11; 95% CI, 1.07-1.15; *P* < .001), a history of alcohol or drug abuse treatment (ARR, 1.90; 95% CI, 1.42-2.53; *P* < .001), and receiving active placebo (ARR, 1.27; 95% CI, 1.03-1.56; *P* = .03). Worst pain reported on postoperative day 10 was the best predictor of time to pain resolution (HR, 0.83; 95% CI, 0.78-0.87; *P* < .001), opioid cessation (HR, 0.84; 95% CI, 0.80-0.89; *P* < .001), and complete surgical recovery (HR, 0.91; 95% CI, 0.86-0.96; *P* < .001).

**Conclusions and Relevance:**

This study has shown a possible uniform predictor of remote postoperative pain, opioid use, and recovery that can be easily assessed. Future work is needed to replicate these findings.

**Trial Registration:**

ClinicalTrials.gov Identifier: NCT01067144

## Key Points

**Question:**

Which prospectively assessed descriptor of the acute pain trajectory in the first 10 days after surgery best estimates the likelihood of remote pain resolution, opioid cessation, and patient-reported complete recovery after surgery?

**Findings:**

In this secondary analysis of a randomized clinical trial of 422 patients, the worst surgical-site pain intensity over the last 24 hours reported on postoperative day 10 appeared to be the best predictor of remote pain resolution, opioid cessation, and complete recovery after surgery.

**Meaning:**

A possibly uniform predictor of disparate surgical outcomes long after hospital discharge may be easily assessed.

## Teaser

This secondary analysis of a randomized clinical trial examines levels of postoperative pain to identify a factor that may be associated with the probability of pain cessation, opioid cessation, and recovery.

## Introduction

The incidence of persistent postsurgical pain (PPSP) varies from 10% to 50%,^1^ and 2% to 10% of patients report severe, chronic pain after surgery.^2^ The severity and duration of postoperative pain are influenced by factors including preexisting pain.^3,4,5,6^ In a meta-analysis of 29 993 patients who had undergone total knee arthroplasty exclusively examining preoperative risk factors, preoperative pain most commonly demonstrated a significant association with PPSP.^7^ In contrast, research simultaneously examining preoperative and postoperative pain as risk factors reported that only acute postoperative pain in the first week after surgery is associated with PPSP among patients undergoing thoracotomy,^3^ total knee arthroplasty,^8^ and breast surgery.^9,10^ In a large European acute postoperative pain registry, the percentage of time of severe pain on postoperative day (POD) 1 across 18 types of surgery was associated with an increased risk of PPSP.^11^ Research is needed to determine which dimensions of acute postoperative pain best estimate the probability PPSP to guide clinical care. More patients experience pain and resulting impaired function after discharge. Early identification of high-risk patients would facilitate targeted referrals to specialists and resource-intensive interventions to prevent the development of PPSP.

The aim of this study was to conduct a secondary analysis of the Stanford Accelerated Recovery Trial (START),^12^ a randomized clinical trial that assessed the effect of perioperative use of gabapentin on postoperative pain resolution and opioid cessation. We aimed to use data-mining algorithms to identify patient clusters based on the first 10 daily pain scores after surgery, explore psychosocial characteristics associated with each cluster, and determine which descriptor of the first 10 postoperative pain scores best estimates the probability of 3 outcomes: remote pain cessation, opioid cessation, and surgical recovery. We hypothesized that patient clusters predictive of poorer remote outcomes would be associated with preoperative psychosocial characteristics. We also hypothesized that high-risk patient clusters would be a superior predictor of outcomes over other acute pain descriptors.

## Methods

### Data Source

This is a secondary analysis of the START,^12^ a randomized, double-blind, placebo-controlled trial of perioperative use of gabapentin in a mixed surgical cohort of patients undergoing eligible operations (thoracotomy, video-assisted thoracoscopic surgery, primary or revision total hip replacement, primary or revision total knee replacement, unilateral or bilateral mastectomy, breast lumpectomy with or without sentinel node biopsy or axillary node dissection, hand surgery, carpal tunnel surgery, knee arthroscopy, shoulder arthroplasty, and shoulder arthroscopy) at a single-center, tertiary referral teaching hospital. A detailed description of the study protocol and trial methods has been published,^12^ and a summary is provided in the eMethods 1 of the Supplement. The eFigure in the Supplement details participant enrollment, randomization, and follow-up. Participants were enrolled between May 25, 2010, and July 25, 2014. Data analysis for the present study was performed from January 1 to August 1, 2018. All aspects of the START protocol, as well as the present analysis, were approved by the Stanford University Institutional Review Board, and all patients provided written, informed consent. The participants did not receive financial compensation. This study followed the Consolidated Standards of Reporting Trials (CONSORT) reporting guideline.

### Assessments

Participants completed a presurgical questionnaire packet that assessed demographics, as well as pain and opioid use with the Brief Pain Inventory.^13^ Investigator-generated measures of self-reported likelihood of developing chronic pain after surgery (4 possible responses: not at all, unlikely, somewhat likely, very likely), self-perceived likelihood of chronic pain after injury (2 possible responses: more likely or less likely), self-perceived sensitivity to pain (3 possible responses: less than average, average, more than average), self-reported prolonged recovery times from past injuries (response to the question, “Have you had unexpectedly long recovery times from injuries in the past?”), and self-perceived likelihood of addiction to pain medication after surgery (not at all, unlikely, somewhat likely, very likely) were included.^14^ The Opioid Risk Tool (range, 0-26: 0-3, low risk; 4-7, moderate risk; ≥8, high risk) was administered to identify patients at risk for opioid-related aberrant behaviors.^15^ Additional assessments included the Marlow-Crowne Social Desirability Scale (range, 0-33: 0-8, low concern for social approval; 20-33, high concern for social approval),^16^ Barratt Impulsivity Scale (range, 30-120; higher scores represent greater impulsiveness),^17^ Posttraumatic Stress Disorder Checklist–Civilian Version (range, 17-85; higher scores represent more self-reported posttraumatic stress disorder symptoms),^18^ State Anxiety Inventory (range,20-80; higher scores represent increasing state of anxiety-anxiety in response to a specific situation),^19^ Trait Anxiety Inventory (range, 20-80; higher scores represent increasing trait anxiety-propensity to experience anxiety),^19^ Beck Depression Inventory-II (range, 0-63; higher scores represent higher depressive symptoms: 0-13, minimal; 14-19, mild; 20-28 moderate; 29-63, severe),^20^ and Euroqol Visual Analog Scale (range from 0-100 for self-assessment of health: 0, worst imaginable health state; 100, best imaginable health sate).^21^

After discharge, a modified Brief Pain Inventory (eMethods 1 in the Supplement) was administered over the phone to assess pain related to the surgical site, medication use, and pain interference. Specifically, patients reported 3 separate pain scores during each telephone call. Patients rated their average and worst pain intensity at the surgical site over the past 24 hours and current pain intensity at the surgical site at the time of assessment. Pain was rated on a 0 (no pain) to 10 (pain as bad as you can imagine) scale per the Numeric Pain Rating Scale. Calls occurred daily for the first 3 months, weekly thereafter up to 6 months, and monthly thereafter up to 2 years after surgery amounting to 19 511 distinct postoperative calls. Calls continued until patients reached pain cessation, opioid cessation, and full recovery up to 2 years after surgery.

### Study Outcomes

Primary outcomes included time to pain resolution (5 consecutive reports of 0 of 10 average pain over the last 24 hours at the surgical site on the Numeric Pain Rating Scale), time to opioid cessation (5 consecutive reports of 0 opioid use over the past 24 hours), and patient-reported complete surgical recovery (responding yes to the question, “Would you say that you are fully recovered from your surgery?”).

### Statistical Analysis

#### Postoperative Acute Pain Cluster Analysis

**Figure 1.**
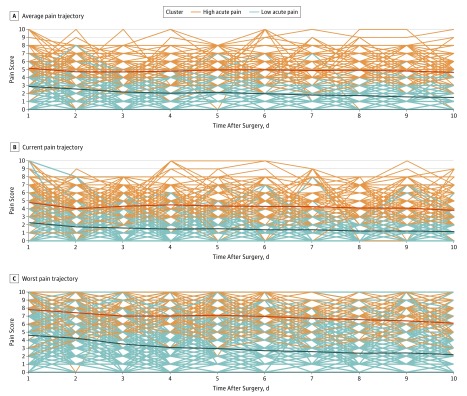
Individual Acute Pain Trajectories Comparing the High vs Low Acute Pain Clusters Each line represents an individual patient’s acute pain trajectory for the average (A), current (B), and worst (C) pain scores reported over the first 10 postoperative days, with the solid bold lines representing the overall cluster trajectory.

Data-mining algorithms were applied using the R programming language, version 3.3 (R Foundation). Each patient reported 3 pain scores (average, current, worst) during each telephone call after surgery. Each category of pain scores (average, current, worst) during the first 10 postoperative days constituted an acute pain trajectory. Thus, each patient had 3 distinct trajectories representing the average, current, and worst reported pain scores. Trajectories within each pain score category were compared for similarity using the k-means clustering algorithm (Figure 1) to assign each patient’s trajectory to a cluster. Patient trajectories were compared in the algorithm through Euclidean distance (the square root of the sum of each dimensional distance squared) between values at each point in time. Details of the method are described in eMethods 2 in the Supplement. Clusters generated by the algorithm were evaluated for distinctness or separation using Calinski-Harabasz scores.^22^

#### Cox Proportional Hazards Regression and Sensitivity Analyses

Subsequent statistical analyses were performed with SAS software, version 9.4 (SAS Institute Inc). The outcomes, time to pain resolution, opioid cessation, and complete surgical recovery were analyzed separately using Cox proportional hazards regression. Given the different underlying hazard rates attributed to different operations, all subsequent analyses were stratified by surgery type.^12^ Fifteen acute pain descriptors, including the average, current, and worst acute pain clusters, mean and median of each set of pain scores over the first 10 postoperative days, and average, current, and worst pain scores reported on POD 10, were evaluated as predictors in univariate analyses of the outcomes. The linear slope of each acute pain trajectory and corresponding goodness-of-fit index (*r*^2^ value) was calculated for each patient and similarly evaluated as a predictor. Model efficiency was determined by goodness-of-fit indices, with lowest Akaike information criteria score indicating the model with the best fit.

#### Modified Poisson Regression Analysis of the Highest-Risk Patient Cluster

Based on the sensitivity analyses, the highest-risk pain cluster was identified. Continuous variables were compared with 2-tailed, unpaired *t* tests, and categorical variables were compared with the χ^2^ tests between the high and low pain clusters of the average acute pain trajectory. Modified Poisson regression analysis was used to determine the preoperative psychosocial characteristics associated with the high pain cluster of the average acute pain trajectory.^23^ This approach allows for estimation of relative risk and 95% CIs with use of robust error variances.^23^ The risk of belonging to the high pain cluster of the average acute pain trajectory was compared with the low-pain cluster. Preoperative explanatory variables considered for inclusion in the model were those hypothesized to affect acute pain, including trial treatment group (gabapentin vs active placebo), sex, age, race/ethnicity, marital status, employment status, disability status, preoperative pain within and outside of the surgical site, author-generated assessments (likelihood of chronic postsurgical pain, chronic postinjury pain, and addiction; and history of prolonged recovery after previous injuries), current opioid use, history of alcohol or drug abuse treatment, and psychosocial assessments (Opioid Risk Tool,^15^ Marlow-Crowne Social Desirability Scale,^16^ Barratt Impulsivity Scale,^17^ Posttraumatic Stress Disorder Checklist–Civilian Version,^18^ State Anxiety Inventory,^19^ Trait Anxiety Inventory,^19^ Beck Depression Inventory-II,^20^ and Euroqol Visual Analog Scale^21^). Model building sensitivity analyses included examination of optimal model fit through automated forward, backward, stepwise, and lasso selection algorithms. Model efficiency was determined by goodness-of-fit indices. Specifically, the lowest Akaike information criterion score indicated the model with the best fit. Testing of collinearity or multicollinearity among predictors included collinearity diagnostics for all variables considered for inclusion in the model.

## Results

### Sample Characteristics

Of the 422 patients who underwent randomization, 410 patients received treatment, provided at least 1 day of follow-up data and were included in the primary START analysis. Thirty-nine patients (9.5%) were excluded from this study on the basis of incomplete pain trajectories (>10% of postoperative pain scores missing from each trajectory). Imputation using last observation carried forward followed by next observation carried backward (for missing values at the beginning of the trajectory) were used to complete the pain trajectories for the remaining 371 patients (90.5%). Of these, 146 (39.4%) were men; mean (SD) age was 56.67 (11.70) years. Table 1 reports the preoperative baseline characteristics for participants in the high vs low pain clusters of the average pain trajectories. A total of 193 patients (52.0%) were partitioned into the low pain cluster and 178 individuals (48.0%) were partitioned into the high pain cluster. Overall, median (interquartile range [IQR]) pain duration in the high vs low pain cluster was 92.0 (50.0-234.0) vs 40.0 (16.0-82.0) days, opioid use was 39.0 (21.0-72.0) vs 12.0 (5.0-32.0) days, and time to patient-reported recovery was 89.0 (41.0-162.0) vs 49.0 (24.0-130.0) days.

**Table 1.** Preoperative Baseline Characteristics of Patients According to Immediate Postoperative Pain Cluster of Average Pain Trajectories

Preoperative Baseline Characteristic	No. (%)^a^	
	Low Pain Cluster	High Pain Cluster
Patients, No.	193	178
Age, mean (SD), y	55.8 (12.5)	57.6 (10.7)
Men	82 (42.7)	64 (36.2)
Marital status		
Never married	11 (6.0)	18 (10.5)
Married	136 (73.5)	122 (71.4)
Living with someone	8 (4.3)	6 (3.5)
Divorced or separated	24 (13.0)	18 (10.5)
Widowed	6 (3.2)	7 (4.1)
Race		
American Indian/Alaska Native	10 (5.5)	4 (2.5)
Asian/Pacific Islander	17 (9.4)	7 (4.3)
White	138 (76.2)	137 (84.6)
African American	8 (4.4)	8 (4.9)
Other	8 (4.4)	6 (3.7)
Ethnicity		
Hispanic or Latino	14 (7.8)	13 (8.0)
Disability claim^b^		
Filed	63 (33.3)	78 (44.8)
Pending	16 (8.8)	29 (17.6)
Employment status		
Full-time	85 (45.7)	64 (37.9)
Part-time	17 (9.1)	18 (10.7)
Unemployed	35 (18.8)	38 (22.5)
Retired	49 (26.3)	49 (29.0)
Treatment group		
Active placebo	86 (44.6)	90 (50.6)
Gabapentin	107 (55.4)	88 (49.4)
Surgery type^c^		
Thoracotomy	7 (3.6)	7 (3.9)
Total knee replacement	43 (22.3)	97 (54.5)
Total hip replacement	48 (24.9)	41 (23.0)
Mastectomy	25 (13.0)	16 (9.0)
Lumpectomy	27 (14.0)	2 (1.1)
VATS	17 (8.8)	12 (6.7)
Hand surgery	15 (7.8)	2 (1.1)
Carpal tunnel surgery	2 (1.0)	1 (0.6)
Knee arthroscopy	2 (1.0)	0
Shoulder arthroplasty	3 (1.6)	0
Shoulder arthroscopy	4 (2.1)	0
Baseline pain, mean (SD)^c^^,^^d^		
At surgical site	4.3 (3.0)	6.2 (3.0)
Other than surgical site	1.9 (2.2)	2.9 (2.5)
Self-perceived likelihood of developing chronic pain after surgery		
Not at all	49 (26.5)	34 (19.8)
Unlikely	106 (57.3)	108 (62.8)
Somewhat likely	27 (14.6)	24 (14.0)
Very likely	3 (1.62)	6 (3.5)
Self-perceived likelihood of developing chronic pain after injury		
More likely	29 (15.7)	29 (17.1)
Less likely	156 (84.3)	141 (82.9)
Self-perceived sensitivity to pain^b^		
More than average	10 (5.4)	22 (13.0)
Average	106 (56.7)	94 (55.6)
Less than average	71 (38.0)	53 (31.4)
History of unexpectedly long recovery times from injuries in the past^b^	22 (11.8)	35 (20.7)
Past 30-d prescription opioid use	13 (6.7)	21 (11.9)
Self-perceived likelihood of addiction to pain medication after surgery^b^		
Not at all	99 (53.5)	88 (51.2)
Unlikely	82 (44.3)	65 (38.4)
Somewhat likely	3 (1.6)	16 (9.3)
Very likely	1 (0.5)	2 (1.2)
Opioid Risk Tool score^e^^,^^f^	1.9 (2.8)	2.8 (3.5)
History of alcohol or drug abuse treatment^b^	4 (2.2)	13 (7.7)
Marlow-Crowne Social Desirability Scale score, mean (SD)^g^	20.3 (5.8)	20.6 (6.0)
Barratt Impulsivity Scale score, mean (SD)^h^	68.7 (5.8)	68.5 (7.7)
PTSD Checklist-Civilian Version score, mean (SD)^i^	24.0 (8.1)	25.7 (9.6)
State Anxiety Inventory score, mean (SD)^b^^,^^j^	33.3 (10.5)	36.4 (11.3)
Trait Anxiety Inventory score, mean (SD)^b^^,^^k^	31.5 (9.9)	34.2 (10.8)
Beck Depression Inventory-ll score, mean (SD)^e^^,^^l^	8.6 (6.2)	10.9 (7.3)
Euroqol VAS, mean (SD)^b^^,^^m^	74.2 (18.8)	68.8 (18.8)
Postoperative, median (IQR), d		
Pain duration	40.0 (16.0-82.0)	92.0 (50.0-234.0)
Opioid use duration	12.0 (5.0-32.0)	39.0 (21.0-72.0)
Time to full recovery	49.0 (24.0-130.0)	89.0 (41.0-162.0)

Abbreviations: IQR, interquartile range; PTSD, posttraumatic stress disorder; VAS, visual analog scale; VATS, video-assisted thoracoscopic surgery.

^a^ Percentages do not include patients with missing data.

^b^ Significant difference between clusters, *P* < .05.

^c^ Significant difference between clusters, *P* < .001.

^d^ Scale range, 1 to 10 (1, no pain; 10, pain as bad as you can imagine).

^e^ Significant difference between clusters, *P* < .005.

^f^ Scale range, 0 to 26 (0-3, low risk; 4-7, moderate risk; ≥8, high risk).

^g^ Scale range, 0 to 33 (0-8, low concern for social approval; 20-33, high concern for social approval).

^h^ Scale range, 30 to 120 (higher scores represent greater impulsiveness).

^i^ Scale range, 17 to 85 (higher scores represent more self-reported PTSD symptoms).

^j^ Scale range, 20 to 80 (higher scores represent increasing state of anxiety-anxiety in response to a specific situation).

^k^ Scale range, 20 to 80 (higher scores represent increasing trait anxiety-propensity to experience anxiety).

^l^ Scale range, 0 to 63 (higher scores represent higher depressive symptoms: 0-13, minimal; 14-19, mild; 20-28 moderate; 29-63, severe).

^m^ Scale range, 0 to 100 for self-assessment of health (0, worst imaginable health state; 100, best imaginable health state).

### Postoperative Acute Pain Cluster Analysis

Clusters within each group of pain trajectories were generated using longitudinal k-means clustering. Intracluster variation and intercluster distinctness were maximized for a 2-cluster solution (Figure 1), with Calinski-Harabasz scores greater than 300. Cluster success scores remained constant throughout each iteration of the algorithm, indicating consistent success in separating patients into similar groupings. High vs low pain clusters were identified within each category of pain trajectories.

### Cox Proportional Hazards Regression and Sensitivity Analyses

**Figure 2.**
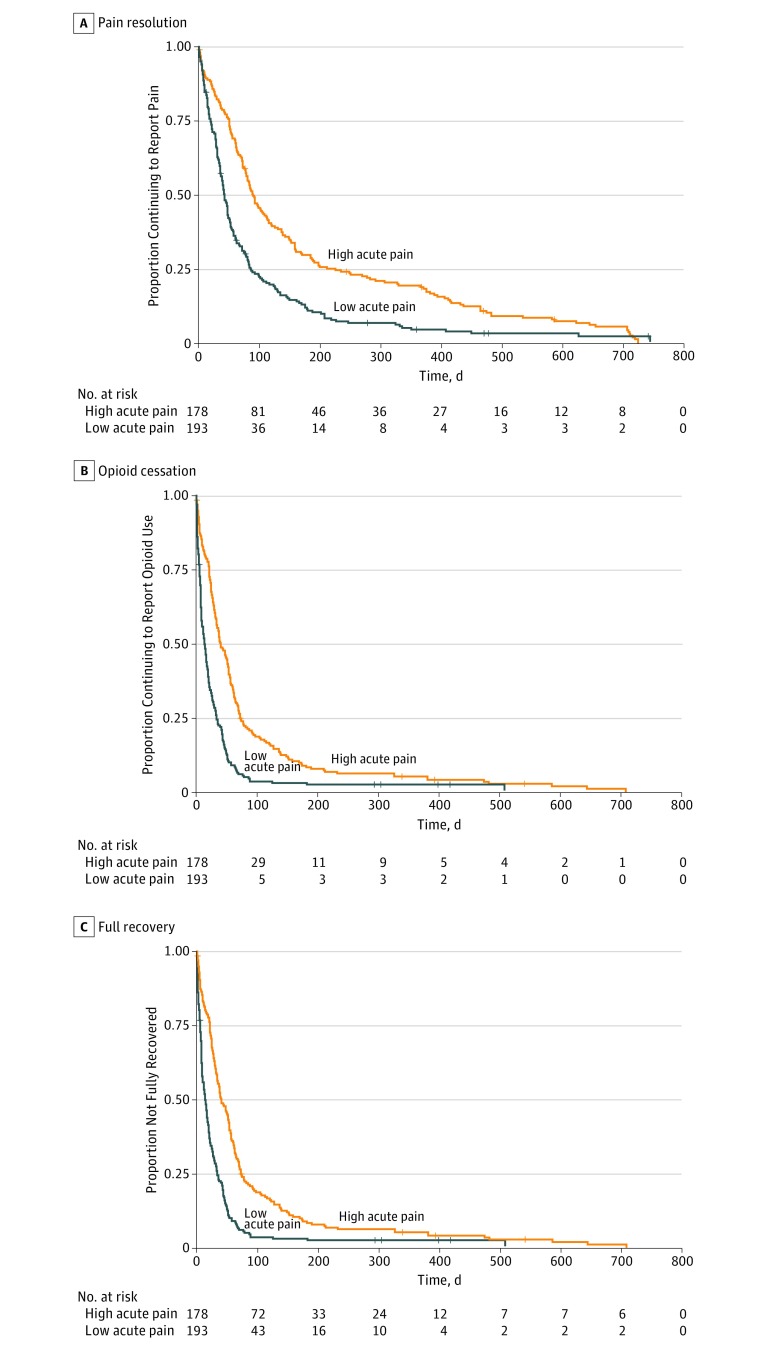
High vs Low Acute Postoperative Pain Clusters of the Average Pain Trajectory and Postoperative Outcomes A, Median time to pain cessation in the high- vs low-risk group was 92 (interquartile range [IQR], 50-234) vs 40 (IQR,16-82) days. Log-rank *P* < .001. B, Median time to opioid cessation in the high- vs low-risk group was 39 (IQR, 21-72) vs 12 (IQR, 5-32) days. Log-rank *P* < .001. C, Median time to full recovery in the high- vs low-risk group was 89 (IQR, 41-162) vs 49 (IQR, 24-130) days. Log-rank *P* = .01.

Following cluster analyses, we examined cluster variables as predictors, using Cox proportional hazards regression and stratification by operation. Figure 2 shows the corresponding Kaplan-Meier curves stratified by pain cluster of the average pain trajectories for all outcomes demonstrating good separation and maintenance of the proportional hazards assumption. Median time to pain cessation in the high- vs low-risk group was 92 days (IQR, 50-234) vs 40 days (IQR, 6-82) (log-rank *P* < .001). Median time to opioid cessation in the high- vs low-risk group was 39 days (IQR, 21-72) vs 12 days (IQR, 5-32) (log-rank *P* < .001). Median time to full recovery in the high- vs low- risk group was 89 days (IQR, 41-162) vs 49 days (IQR, 24-130) (log-rank *P* = .01). Table 2 lists the corresponding hazard ratios (HRs) for all outcomes examining the high vs low pain clusters within each pain trajectory category. The high pain clusters within each trajectory category significantly predicted time to pain (eg, high vs low pain cluster of average pain trajectories: time to pain cessation, HR, 0.63; 95% CI, 0.50-0.80; *P*<.001) and opioid cessation (eg, high vs low pain cluster of average pain trajectories: time to opioid cessation, HR, 0.52; 95% CI, 0.41-0.67; *P* < .001), but was not a predictor of time to surgical recovery (HR, 0.89; 95% CI, 0.69-1.14; *P* = .89).

**Table 2.** Acute Pain Clusters as Determinants of Remote Pain Cessation, Opioid Cessation, and Full Recovery After Surgery

Outcome	HR (95% CI)	*P* Value
**High vs Low Pain Cluster of Average Pain Trajectories**^**a**^		
Time to pain cessation	0.63 (0.50-0.80)	<.001
Time to opioid cessation	0.52 (0.41-0.67)	<.001
Time to full recovery	0.89 (0.69-1.14)	.89
**High vs Low Pain Cluster of Current Pain Trajectories**^a^		
Time to pain cessation	0.71 (0.56-0.89)	.003
Time to opioid cessation	0.66 (0.52-0.83)	<.001
Time to full recovery	1.01 (0.79-1.28)	.97
**High vs Low Pain Cluster of Worst Pain Trajectories**^a^		
Time to pain cessation	0.69 (0.54-0.89)	.004
Time to opioid cessation	0.57 (0.44-0.73)	<.001
Time to full recovery	1.04 (0.79-1.36)	.78

Abbreviation: HR, hazard ratio.

^a^ Stratification by type of surgery.

Sensitivity analyses examined descriptors of acute pain, including the pain clusters of the average, current, and worst pain trajectories; mean and median of the average, current, and worst pain scores during the first 10 postoperative days; average, current, and worst pain scores on POD 10; and linear slopes of the pain trajectories. Table 3 lists the results of the sensitivity analyses. Overall, worst surgical-site pain over the past 24 hours reported on POD 10 was the best predictor of remote time to pain cessation (HR, 0.83; 95% CI, 0.78-0.87; *P* < .001), time to opioid cessation (HR, 0.84; 95% CI, 0.80-0.89; *P* < .001), and time to complete surgical recovery (HR, 0.91; 95% CI, 0.86-0.96; *P* < .001). Less than 40% of the variance in any given patient’s acute pain trajectory was explained by a linear model (average pain slope median *r*^2^ = 0.28, interquartile range [IQR] 0.07-0.58; worst pain slope median *r*^2^ = 0.33, IQR, 0.12-0.63; current pain slope median *r*^2^ = 0.25, IQR, 0.07-0.46). The linear slope was the least predictive of pain resolution and opioid cessation but was more predictive than the pain clusters for time to recovery. Additional results regarding optimal cutoff values of the worst pain score on POD 10 are included in the eAppendix in the Supplement.

**Table 3.** Univariate Sensitivity Analyses of Acute Pain Descriptors as Determinants of Remote Pain Cessation, Opioid Cessation, and Full Recovery After Surgery

Predictor	HR (95% CI)	*P* Value	AIC
**Sensitivity Analysis for Time to Pain Cessation**^**a**^			
Postoperative day 10 pain score			
Worst	0.83 (0.78-0.87)	<.001	1843.229
Average	0.81 (0.76-0.87)	<.001	1855.158
Current	0.87 (0.82-0.93)	<.001	1872.931
Worst pain scores over 10 d			
Median	0.88 (0.83-0.94)	<.001	2061.154
Mean	0.87 (0.83-0.93)	<.001	2061.725
Average pain scores over 10 d			
Mean	0.87 (0.80-0.95)	.001	2067.125
Median	0.89 (0.82-0.96)	.004	2069.435
Current pain scores over 10 d			
Mean	0.91 (0.84-0.99)	.02	2072.804
Median	0.93 (0.86-1.00)	.04	2073.811
High pain cluster of pain trajectories			
Average	0.63 (0.50-0.80)	<.001	2315.394
Current	0.71 (0.56-0.89)	.003	2320.682
Worst	0.69 (0.54-0.89)	.004	2321.204
Linear slope of the pain trajectories			
Worst	0.68 (0.46-1.00)	.05	2325.657
Average	0.70 (0.44-1.12)	.14	2327.304
Current	0.83 (0.54-1.28)	.40	2328.814
**Sensitivity Analysis for Time to Opioid Cessation**^**a**^			
Postoperative day 10 pain score			
Worst	0.84 (0.80-0.89)	<.001	1883.381
Average	0.82 (0.76-0.88)	<.001	1894.527
Current	0.85 (0.79-0.91)	<.001	1903.222
Worst pain scores over 10 d			
Mean	0.85 (0.79-0.90)	<.001	2087.508
Median	0.86 (0.82-0.91)	<.001	2089.049
Average pain scores over 10 d			
Mean	0.85 (0.79-0.92)	<.001	2098.918
Median	0.87 (0.80-0.93)	<.001	2099.983
Current pain scores over 10 d			
Mean	0.87 (0.81-0.95)	.001	2104.030
Median	0.89 (0.83-0.96)	.002	2105.688
High pain cluster of pain trajectories			
Average	0.52 (0.41-0.67)	<.001	2342.622
Worst	0.57 (0.44-0.73)	<.001	2351.640
Current	0.66 (0.52-0.83)	<.001	2368.149
Linear slope of the pain trajectories			
Average	0.77 (0.47-1.26)	.30	2369.041
Worst	0.81 (0.55-1.21)	.30	2369.094
Current	0.81 (0.51-1.27)	.36	2369.313
**Sensitivity Analysis for Time to Recovery**^**a**^			
Postoperative day 10 pain score			
Worst	0.91 (0.86-0.96)	<.001	1671.220
Average	0.89 (0.83-0.95)	.001	1672.025
Current	0.93 (0.87-0.99)	.03	1678.362
Worst pain scores over 10 d			
Mean	1.01 (0.94-1.07)	.88	1868.195
Median	1.00 (0.95-1.06)	.90	1868.201
Average pain scores over 10 d			
Median	1.02 (0.95-1.10)	.64	1867.996
Mean	1.02 (0.94-1.10)	.66	1868.023
Current pain scores over 10 d			
Mean	1.04 (0.96-1.13)	.31	1867.194
Median	1.03 (0.96-1.11)	.46	1867.668
High pain cluster of pain trajectories			
Average	0.89 (0.69-1.14)	.36	2066.712
Worst	1.04 (0.79-1.36)	.78	2067.480
Current	1.01 (0.79-1.28)	.97	2067.559
Linear slope of the pain trajectories			
Average	0.64 (0.37-1.11)	.11	2065.001
Current	0.69 (0.41-1.16)	.16	2065.631
Worst	0.78 (0.52-1.18)	.24	2066.192

Abbreviations: AIC, Akaike information criterion; HR, hazard ratio.

^a^ Stratification by type of surgery.

### Modified Poisson Regression Analysis

Because clustering of the average pain trajectories was the best predictor of time to opioid and pain cessation, we compared preoperative characteristics of participants between the high and low pain clusters of the average pain trajectories. There was a low chance of collinearity when examining variables considered for inclusion in the final model (tolerance range, 0.34-0.92; variance inflation factor range, 1.09-2.97). Several preoperative risk factors emerged for categorization to the high acute pain cluster after surgery (eTable in the Supplement). These factors included female sex (adjusted relative risk [ARR], 1.36; 95% CI, 1.08-1.70; *P* = .008), elevated preoperative pain at the future surgical site (ARR, 1.11; 95% CI, 1.07-1.15; *P* < .001), a history of alcohol or drug abuse treatment (ARR, 1.90; 95% CI, 1.42-2.53; *P* < .001), and randomization to active placebo (ARR, 1.27; 95% CI, 1.03-1.56; *P* = .03).

## Discussion

We identified high vs low pain clusters within trajectories of the first 10 postoperative daily pain scores (reported as average, current, and worst pain) in a mixed surgical cohort. Patients categorized to each of 3 high pain clusters had longer durations of pain and opioid use regardless of the operation performed. Of the high pain clusters, the cluster for the average pain trajectory was the best predictor of time to pain and opioid cessation. Several preoperative characteristics correlated with assignment to the high pain cluster, including worse baseline pain at the future surgical site, a history of alcohol or drug abuse treatment, and randomization to the active placebo group. We also identified a sex difference, noting that women were at increased risk of categorization to the high pain cluster of average pain trajectories, reflective of prior work demonstrating higher pain trajectories in the first 24 hours after surgery.^24^

Previous studies had not identified uniform preoperative predictors of remote pain and opioid cessation after surgery.^14,25^ We identified worst pain over the past 24 hours reported on POD 10 as a significant immediate postoperative predictor of remote pain resolution, opioid cessation, and complete surgical recovery. This measure was a better predictor of all 3 outcomes compared with 14 other descriptors of acute pain over the first 10 postoperative days. The simplicity of this measure applied to current clinical practice could possibly facilitate early identification of patients requiring more-intensive, interdisciplinary, postoperative pain management. In addition, decisions to continue or discontinue multimodal analgesia protocols might be aided by this immediate postoperative pain measure. At the present, preliminary evidence suggests that patients with severe postoperative pain and high opioid requirements may benefit from referral to an outpatient, follow-up, acute pain service for facilitation of continued multimodal analgesia, postoperative opioid tapering, and referral for multidisciplinary pain resources.^26^ Our immediate postoperative predictor may facilitate identification and referral of high-risk patients through these transitional pain services.

Our study possesses a number of strengths. In particular, the frequency of assessments provides particular granularity with an association with each outcome. Our outcomes were captured through 19 511 distinct postoperative telephone calls. Also, the extended duration of postoperative follow-up to 2 years after surgery accounted for natural variations in surgical recovery. Rather than relying on assessments at discreet, predetermined postoperative times (eg, 3, 6, 12 months), we captured sustained remission of pain and opioid use. As evidenced by individual pain trajectories, the general assumption that pain intensity decreases linearly month to month does not reflect true day-to-day variation in pain intensity.

Our findings extend previous work examining acute postoperative pain as a predictor of PPSP. Prior work has characterized diverse pain trajectories over the first 6 days after surgery using a linear fit of acute postoperative pain modeling.^27^ In contrast, acute pain trajectories in our work revealed excessive variation over the first 10 postoperative days. Approximating a patient’s acute pain trajectory using a linear assumption explained less than 40% of the variance in a patient’s trajectory. Thus, we identified a novel technique of patient clustering negating the need for linear model fitting. This approach allows for natural variations in the acute pain trajectory likely resulting from important pain-trajectory mediating events. For example, pain may acutely increase after hospital discharge secondary to increased physical activity, discontinuation of regional anesthetic techniques, or suboptimal analgesic dosing. Thomazeau et al^28^ characterized the acute postoperative pain trajectory over the first 5 days as a predictor of PPSP in patients undergoing knee arthroplasty. Similar to our findings, high and low pain intensity acute pain trajectories were identified, and categorization to the high pain trajectory was associated with nonzero pain intensity 6 months after surgery.^28^ In contrast, prior research reported that pain trajectories over the first 4 postoperative days were not associated with pain intensity 6 months after total hip arthroplasty.^29^ The difference in findings is likely owing to use of growth mixture modeling,^29^ which entails numerous assumptions regarding both within-person and random effects. Thus, results can be sensitive to these assumptions.^30^ Also, assumptions or model fit can be difficult to check. Our work identified high vs low acute pain clusters as predictive of both pain resolution and opioid cessation in a diverse surgical cohort. Given that preoperative opioid use was not associated with categorization to the high pain cluster, it is likely that the high pain cluster is characterizing a unique acute pain response. Our findings add to research examining acute postoperative pain as a predictor of PPSP by not only examining acute pain clustering variables as a predictor of remote pain cessation, opioid cessation, and surgical recovery, but also comparing these variables with many other descriptors of the acute pain trajectory. Contrary to our hypothesis, the acute pain clustering variables were not the superior predictor. Future work to replicate our findings is needed to confirm worst pain reported on POD 10 as a significant immediate postoperative predictor of remote outcomes.

### Limitations

Our study has limitations. As a secondary analysis, the data originate from a clinical trial, and future work to replicate these study findings are needed across a variety of surgical cohorts. Although categorization to the high pain cluster of the average acute pain trajectory was predictive of remote pain and opioid cessation, worst pain reported on POD 10 was predictive of all outcomes (persistent pain, prolonged opioid use, and delayed surgical recovery). External validation in separate surgical cohorts is needed to address the possibility of overfitting predictors to this specific cohort of patients. Future studies should seek to replicate the categorization of distinct surgical cohorts into high vs low acute pain clusters and determine the best acute pain predictors of remote outcomes. Also, the trial did not dictate a standard postoperative pain management protocol, and treatment with opioids was not actively discontinued or tapered after surgery. Given the ongoing opioid crisis and efforts to limit opioid prescribing for acute pain, it will be important to validate our findings in future surgical cohorts.

Randomization to active placebo was a risk factor for categorization to the high pain cluster of the average acute pain trajectory over the first 10 postoperative days in our cohort. Contrasting with the original START findings, which did not identify any differences in treatment group on remote pain resolution (5 consecutive postoperative reports of 0 of 10 levels of average pain at the surgical site on the Numeric Pain Rating Scale). Median time to pain resolution in the START was 84 days (IQR, 36-203) in patients receiving gabapentin and 73 days (IQR, 36-231) in patients receiving active placebo.^12^ Only 9.6% of patients who had received gabapentin and 5.6% of patients who had received active placebo had reached pain cessation by POD 10.^12^ Because both perioperative gabapentin and active placebo were administered up to 72 hours after surgery, treatment may have been associated with reducing the intensity of acute postoperative pain during the first 10 postoperative days, affecting categorization to the high vs low acute pain cluster rather than remote pain resolution originally reported in START.

Another limitation of the findings centers on the duration of the immediate postoperative pain trajectories. We arbitrarily examined the first 10 postoperative pain scores. However, it is unclear whether earlier postoperative assessment would allow for similar estimation of the probability of remote outcomes. Future work to determine the critical period for assessment of immediate postoperative pain is needed. If earlier predischarge assessment of worst postoperative pain over the past 24 hours affords comparable predictability of remote outcomes, interventions could be initiated before hospital discharge with closer follow-up of vulnerable patients. Also, earlier assessment would allow for preemptive, predischarge referral to specialist care and transitional pain services.

## Conclusions

This study’s findings suggest that the worst surgical-site pain over the past 24 hours reported on POD 10 was a significant immediate postoperative predictor of remote pain resolution, opioid cessation, and complete surgical recovery months after surgery, and this measure was probably a better predictor of long-term outcomes than 14 alternative descriptors of the acute pain trajectory in our cohort. This measure can be assessed in person or over the telephone to identify patients at high risk for delayed recovery, prolonged opioid use, and PPSP. Future work to replicate these findings is needed to inform clinical care. Ultimately, early identification of high-risk patients would facilitate personalized care with closer follow-up, earlier referral for specialist care, and extension of multimodal pain regimens.

## Notes

Supplement. **eMethods 1.** Design and Modified Brief Pain Inventory **eMethods 2.** Statistical Analysis **eAppendix.** Detailed Findings **eFigure.** Enrollment, Randomization, and Follow-up for the START Trial **eTable.** High Pain Cluster of Average Pain Trajectories Preoperative Risk Factors

## References

[1] Chapman CR, Vierck CJ The transition of acute postoperative pain to chronic pain: an integrative overview of research on mechanisms. J Pain. 2017;18(4):-. doi:10.1016/j.jpain.2016.11.004 27908839

[2] Kehlet H, Jensen TS, Woolf CJ Persistent postsurgical pain: risk factors and prevention. Lancet. 2006;367(9522):1618-1625. doi:10.1016/S0140-6736(06)68700-X 16698416

[3] Gottschalk A, Ochroch EA Clinical and demographic characteristics of patients with chronic pain after major thoracotomy. Clin J Pain. 2008;24(8):708-716. doi:10.1097/AJP.0b013e318174badd 18806536

[4] Maguire MF, Ravenscroft A, Beggs D, Duffy JP A questionnaire study investigating the prevalence of the neuropathic component of chronic pain after thoracic surgery. Eur J Cardiothorac Surg. 2006;29(5):800-805. doi:10.1016/j.ejcts.2006.02.002 16581259

[5] Meretoja TJ, Andersen KG, Bruce J, Clinical prediction model and tool for assessing risk of persistent pain after breast cancer surgery. J Clin Oncol. 2017;35(15):1660-1667. doi:10.1200/JCO.2016.70.3413 28524782

[6] Gotoda Y, Kambara N, Sakai T, Kishi Y, Kodama K, Koyama T The morbidity, time course and predictive factors for persistent post-thoracotomy pain. Eur J Pain. 2001;5(1):89-96. doi:10.1053/eujp.2001.0225 11394926

[7] Lewis GN, Rice DA, McNair PJ, Kluger M Predictors of persistent pain after total knee arthroplasty: a systematic review and meta-analysis. Br J Anaesth. 2015;114(4):551-561. doi:10.1093/bja/aeu441 25542191

[8] Puolakka PA, Rorarius MG, Roviola M, Puolakka TJ, Nordhausen K, Lindgren L Persistent pain following knee arthroplasty. Eur J Anaesthesiol. 2010;27(5):455-460. doi:10.1097/EJA.0b013e328335b31c 20299989

[9] Wang L, Guyatt GH, Kennedy SA, Predictors of persistent pain after breast cancer surgery: a systematic review and meta-analysis of observational studies. CMAJ. 2016;188(14):E352-E361. doi:10.1503/cmaj.151276 27402075 PMC5047835

[10] Poleshuck EL, Katz J, Andrus CH, Risk factors for chronic pain following breast cancer surgery: a prospective study. J Pain. 2006;7(9):626-634. doi:10.1016/j.jpain.2006.02.007 16942948 PMC6983301

[11] Fletcher D, Stamer UM, Pogatzki-Zahn E, ; euCPSP Group for the Clinical Trial Network Group of the European Society of Anaesthesiology Chronic postsurgical pain in Europe: an observational study. Eur J Anaesthesiol. 2015;32(10):725-734. doi:10.1097/EJA.0000000000000319 26241763

[12] Hah J, Mackey SC, Schmidt P, Effect of perioperative gabapentin on postoperative pain resolution and opioid cessation in a mixed surgical cohort: a randomized clinical trial. JAMA Surg. 2018;153(4):303-311. doi:10.1001/jamasurg.2017.4915 29238824 PMC5933381

[13] Keller S, Bann CM, Dodd SL, Schein J, Mendoza TR, Cleeland CS Validity of the Brief Pain Inventory for use in documenting the outcomes of patients with noncancer pain. Clin J Pain. 2004;20(5):309-318. doi:10.1097/00002508-200409000-00005 15322437

[14] Carroll I, Barelka P, Wang CK, A pilot cohort study of the determinants of longitudinal opioid use after surgery. Anesth Analg. 2012;115(3):694-702. 22729963 10.1213/ANE.0b013e31825c049f

[15] Webster LR, Webster RM Predicting aberrant behaviors in opioid-treated patients: preliminary validation of the Opioid Risk Tool. Pain Med. 2005;6(6):432-442. doi:10.1111/j.1526-4637.2005.00072.x 16336480

[16] Crowne DP, Marlowe D A new scale of social desirability independent of psychopathology. J Consult Psychol. 1960;24:349-354. doi:10.1037/h0047358 13813058

[17] Patton JH, Stanford MS, Barratt ES Factor structure of the Barratt impulsiveness scale. J Clin Psychol. 1995;51(6):768-774. doi:10.1002/1097-4679(199511)51:6<768::AID-JCLP2270510607>3.0.CO;2-1 8778124

[18] Ruggiero KJ, Del Ben K, Scotti JR, Rabalais AE Psychometric properties of the PTSD Checklist–Civilian Version. J Trauma Stress. 2003;16(5):495-502. doi:10.1023/A:1025714729117 14584634

[19] Spielberger CD State-Trait anxiety Inventory: A Comprehensive Bibliography. 2nd ed Palo Alto, CA: Consulting Psychologists Press; 1989.

[20] Beck AT, Steer RA, Ball R, Ranieri W Comparison of Beck Depression Inventories-IA and -II in psychiatric outpatients. J Pers Assess. 1996;67(3):588-597. doi:10.1207/s15327752jpa6703_13 8991972

[21] EuroQol Group EuroQol—a new facility for the measurement of health-related quality of life. Health Policy. 1990;16(3):199-208. doi:10.1016/0168-8510(90)90421-9 10109801

[22] Liu Y, Li Z, Xiong H, Gao X, Wu J, Wu S Understanding and enhancement of internal clustering validation measures. IEEE Trans Cybern. 2013;43(3):982-994. doi:10.1109/TSMCB.2012.2220543 23193245

[23] Zou G A modified Poisson regression approach to prospective studies with binary data. Am J Epidemiol. 2004;159(7):702-706. doi:10.1093/aje/kwh090 15033648

[24] Tighe PJ, Le-Wendling LT, Patel A, Zou B, Fillingim RB Clinically derived early postoperative pain trajectories differ by age, sex, and type of surgery. Pain. 2015;156(4):609-617. doi:10.1097/01.j.pain.0000460352.07836.0d 25790453 PMC4367128

[25] Carroll IR, Hah JM, Barelka PL, Pain duration and resolution following surgery: an inception cohort study. Pain Med. 2015;16(12):2386-2396. doi:10.1111/pme.12842 26179223 PMC4706803

[26] Tiippana E, Hamunen K, Heiskanen T, Nieminen T, Kalso E, Kontinen VK New approach for treatment of prolonged postoperative pain: APS Out-Patient Clinic. Scand J Pain. 2016;12:19-24. doi:10.1016/j.sjpain.2016.02.008 28850486

[27] Chapman CR, Donaldson GW, Davis JJ, Bradshaw DH Improving individual measurement of postoperative pain: the pain trajectory. J Pain. 2011;12(2):257-262. doi:10.1016/j.jpain.2010.08.005 21237721 PMC3052945

[28] Thomazeau J, Rouquette A, Martinez V, Predictive factors of chronic post-surgical pain at 6 months following knee replacement: influence of postoperative pain trajectory and genetics. Pain Physician. 2016;19(5):E729-E741. 27389116

[29] Pagé MG, Katz J, Curtis K, Lutzky-Cohen N, Escobar EM, Clarke HA Acute pain trajectories and the persistence of post-surgical pain: a longitudinal study after total hip arthroplasty. J Anesth. 2016;30(4):568-577. doi:10.1007/s00540-016-2183-4 27155598

[30] Curran PJ, Obeidat K, Losardo D Twelve frequently asked questions about growth curve modeling. J Cogn Dev. 2010;11(2):121-136. doi:10.1080/15248371003699969 21743795 PMC3131138

