# Clarification of Disclosure

**DOI:** 10.1001/jamanetworkopen.2022.9747

**Published:** 2022-04-06

In the Original Investigation titled “Factors Associated With Acute Pain Estimation, Postoperative Pain Resolution, Opioid Cessation, and Recovery: Secondary Analysis of a Randomized Clinical Trial,”^1^ published March 1, 2019, the conflict of interest disclosure statement should have read as follows: “Dr Mackey reported receiving funding from the Redlich Pain Endowment; serving as president of the American Academy of Pain Medicine from 2014-2015, as a member of the American Society of Anesthesiologists Pain Committee from 2008-2019, and as a member of the advisory board of the American Chronic Pain Association (a nonprofit organization focused on patient education about pain), and reported receiving no compensation from these entities; and also reported receiving travel reimbursement from the American Academy of Pain Medicine, Tarsus Group, Neurovations, International Modulation Society, and FDA-ACCTION to present pain research findings.” This article has been corrected.^1^
