# Supplementary material for: Factors Associated With Acute Pain Estimation, Postoperative Pain Resolution, Opioid Cessation, and Recovery: Secondary Analysis of a Randomized Clinical Trial
Source: JAMA Netw Open. 2019 Mar 1;2(3):e190168. doi: 10.1001/jamanetworkopen.2019.0168 (PMC6484627; doi:10.1001/jamanetworkopen.2019.0168)
Supplement: Supplement. — eMethods 1. Design and Modified Brief Pain Inventory eMethods 2. Statistical Analysis eAppendix. Detailed Findings eFigure. Enrollment, Randomization, and Follow-up for the START Trial eTable. High Pain Cluster of Average Pain Trajectories Preoperative Risk Factors [file jamanetwopen-2-e190168-s001.pdf]

Supplementary Online Content

Hah JM, Cramer E, Hilmoe H, et al. Factors associated with acute pain estimation, postoperative pain resolution, opioid cessation, and recovery: secondary analysis of a randomized clinical trial. *JAMA Netw Open*. 2019;2(3):e190168. doi:10.1001/jamanetworkopen.2019.0168

eMethods 1. Design and Modified Brief Pain Inventory

eMethods 2. Statistical Analysis

eAppendix. Detailed Findings

eFigure. Enrollment, Randomization, and Follow-up for the START Trial

eTable. High Pain Cluster of Average Pain Trajectories Preoperative Risk Factors

This supplementary material has been provided by the authors to give readers additional information about their work.

eMethods 1. Design and Modified Brief Pain Inventory

In summary, English-speaking patients, aged 18 to 75 years-old scheduled for an eligible operation (thoracotomy, video-assisted thoracoscopic surgery, primary or revision total hip replacement, primary or revision total knee replacement, unilateral or bilateral mastectomy, breast lumpectomy with or without sentinel node biopsy or axillary node dissection) were screened and enrolled from May 25, 2010 to July 25, 2014 and followed for 2 years postoperatively. The following operations were added to the protocol mid-study to promote patient recruitment: hand surgery, carpal tunnel surgery, knee arthroscopy, shoulder arthroplasty, and shoulder arthroscopy.

Exclusion criteria were known kidney disease, current gabapentin or pregabalin use, cognitive impairment, history of excessive sedation or adverse reaction to gabapentin, coexisting chronic pain (severity level of >4 of 10 on a numeric rating scale of pain score anywhere, with 10 the most severe level, excluding the future surgical site), conditions precluding postoperative follow-up, suicidality assessed by the Beck Depression Inventory-II (scale range, 0-63, with 0-13 indicating minimal depression; 14-19 mild depression; 20-28 moderate depression; and 29-63 severe depression), pregnancy, ataxia, dizziness, sedation, narrow-angle glaucoma, severe respiratory insufficiency, history of gastric bypass surgery, and obstructive sleep apnea requiring a continuous positive airway pressure device.

2 weeks before surgery, patients were randomized using blocked, stratified randomization by operation and surgeon after study enrollment by research staff. A computer-generated randomization list with corresponding randomization log sheets were provided to the operating room pharmacy by the research team. One log sheet

© 2019 Hah JM et al. *JAMA Network Open.*

was generated per combination of surgeon and operation. The pharmacist documented the patient's information on a randomization card that was placed in a sealed envelope indicating group allocation. Participants, clinicians, and the research staff were blinded to allocation until completion of the primary statistical analyses.

208 patients were randomized to receive gabapentin 1200mg preoperatively and 600mg three times daily postoperatively, and 202 patients were randomized to receive active placebo (lorazepam 0.5mg) and inactive placebo three times daily postoperatively for 72 hours. Participants completed a pre-surgical questionnaire packet assessing pain, opioid use, substance use, and psychosocial variables. Demographics including race and ethnicity were reported by participants.

After surgery, a modified Brief Pain Inventory was administered over the telephone to assess postoperative pain, medication use, and pain interference. Calls occurred daily for the first 3 months, weekly thereafter up to 6 months, and monthly thereafter up to 2 years after surgery amounting to 19,511 distinct postoperative calls. Administration of perioperative gabapentin had no effect on postoperative pain resolution but had a modest effect on promoting opioid cessation after surgery. Following a preplanned interim analysis, the study was stopped early for meeting a futility stopping boundary with regard to the primary end point (time to pain cessation) for the clinical trial.

© 2019 Hah JM et al. *JAMA Network Open.*

Modified Brief Pain Inventory

Please rate your pain from zero to ten where zero is no pain and ten is the worst pain you can imagine on the AVERAGE in the last 24 hours.

	0	1	2	3	4	5	6	7	8	9	10
No Pain											Pain As Bad As You Can Imagine

Please rate your pain from zero to ten where zero is no pain and ten is the worst pain you can imagine right NOW.

	0	1	2	3	4	5	6	7	8	9	10
No Pain											Pain As Bad As You Can Imagine

Please rate your pain from zero to ten where zero is no pain and ten is the worst pain you can imagine at its WORST in the last 24 hours.

	0	1	2	3	4	5	6	7	8	9	10
No Pain											Pain As Bad As You Can Imagine

In the last 24 hours, what medications did you take for your pain?

How many have you taken in the past 24 hours?

In the last 24 hours, how much relief has pain medication provided?

	0	1	2	3	4	5	6	7	8	9	10
No Relief											Complete Relief

In the last 24 hours, have you needed to take your pain medication for any reason other than just pain, for example to help sleep, to reduce anxiety, or to improve mood?

___Yes ____NO

If yes for what?____

In the last 24 hours, has it been necessary for you to take any more pain medication than recommended?

___Yes ____NO

In general, over the last 24 hours which of these options best describes how often you have thought about your pain or pain medication:

Less than once per hour   Several times an hour   Every few minutes   Constantly

Rate the severity of any pain medication side effects you have experienced over the last 24 hours?

	0	1	2	3	4	5	6	7	8	9	10
No Side effects											Most severe side effects You Can Imagine

© 2019 Hah JM et al. *JAMA Network Open.*

On a zero to ten scale where zero is “no interference” and ten is “complete interference” Please rate, during the past 24 hours, how pain has interfered with your:

General Activity

	0	1	2	3	4	5	6	7	8	9	10
Does Not Interfere											Completely Interferes

Mood

	0	1	2	3	4	5	6	7	8	9	10
Does Not Interfere											Completely Interferes

Sleep

	0	1	2	3	4	5	6	7	8	9	10
Does Not Interfere											Completely Interferes

Do you consider yourself to have completely recovered from your surgery?
__yes ___no

If you worked before surgery have you returned to work (whether paid or not--any vocational activity)?
__yes ___no ___not applicable

If unemployed, retired, or permanently no longer part of the work force before surgery, have you returned to your pre-surgery level of activity?
__yes ___no ___not applicable

© 2019 Hah JM et al. *JAMA Network Open.*

eMethods 2. Statistical Analysis

In a given dataset, the k-means algorithm groups observations in the data into a few cohesive clusters by calculating similarities between the different dimensions for each observation (measured with Euclidean distance).[30] The goal of the algorithm is to assign a label or group name for each observation (in this case, each patient) and to predict the center of each group.[31] First, the algorithm initializes the centers for a specified (k) number of clusters randomly. For each observation, the Euclidean distance to each of the k cluster centers is calculated, and the observation is assigned to the cluster with minimum distance. Then, the center (or average observation) of a cluster is recalculated based on the observations that are assigned to that cluster. The process is repeated until the labels for each observation no longer change with each iteration.

© 2019 Hah JM et al. *JAMA Network Open.*

eAppendix. Detailed Findings

We conducted a receiver operating characteristic (ROC) curve analysis of persistent pain 90 days after surgery. Worst pain reported on postoperative day #10 was significantly associated with persistent pain 90 days after surgery (OR 1.48, 95% CI 1.33-1.65, p-value<0.001). Overall, worst pain over the last 24 hours reported on postoperative day #10 was predictive of persistent pain 90 days after surgery (AUC=0.77, 95%CI 0.72-0.82, p-value<0.001). Based on the optimal Youden's J value, patients reporting a worst pain score on postoperative day #10 ≥4 will have an 82.9% chance of having persistent pain 90 days after surgery. Conversely, patients reporting a worst pain score on postoperative day #10 <4 will have 56.3 % chance of not developing persistent pain 90 days after surgery.

Additionally, we conducted a receiver operating characteristic (ROC) curve analysis of persistent opioid use 90 days after surgery. Worst pain reported on postoperative day #10 was significantly associated with persistent opioid use 90 days after surgery (OR 1.61, 95% CI 1.35-1.90, p-value<0.001). Overall, worst pain over the last 24 hours reported on postoperative day #10 was predictive of persistent opioid use 90 days after surgery (AUC=0.81, 95%CI 0.74-0.89, p-value<0.001). Based on the optimal Youden's J value, patients reporting a worst pain score on postoperative day #10 ≥7 will have a 69.7% chance of having persistent opioid use 90 days after surgery. Conversely, patients reporting a worst pain score on postoperative day #10 <7 will have 79.3 % chance of not developing persistent opioid use 90 days after surgery.

© 2019 Hah JM et al. *JAMA Network Open.*

We then conducted a receiver operating characteristic (ROC) curve analysis of delayed recovery 90 days after surgery. Worst pain reported on postoperative day #10 was significantly associated with delayed recovery 90 days after surgery (OR 1.32, 95% CI 1.20-1.45, p-value<0.001). Overall, worst pain over the last 24 hours reported on postoperative day #10 was predictive of delayed recovery 90 days after surgery (AUC=0.70, 95%CI 0.64-0.76, p-value<0.001). Based on the optimal Youden's J value, patients reporting a worst pain score on postoperative day #10 ≥5 will have a 65.0% chance of having delayed recovery 90 days after surgery. Conversely, patients reporting a worst pain score on postoperative day #10 <5 will have 64.6% chance of not developing persistent opioid use 90 days after surgery.

© 2019 Hah JM et al. *JAMA Network Open.*

eFigure. Enrollment, Randomization, and Follow-up for the START trial

.

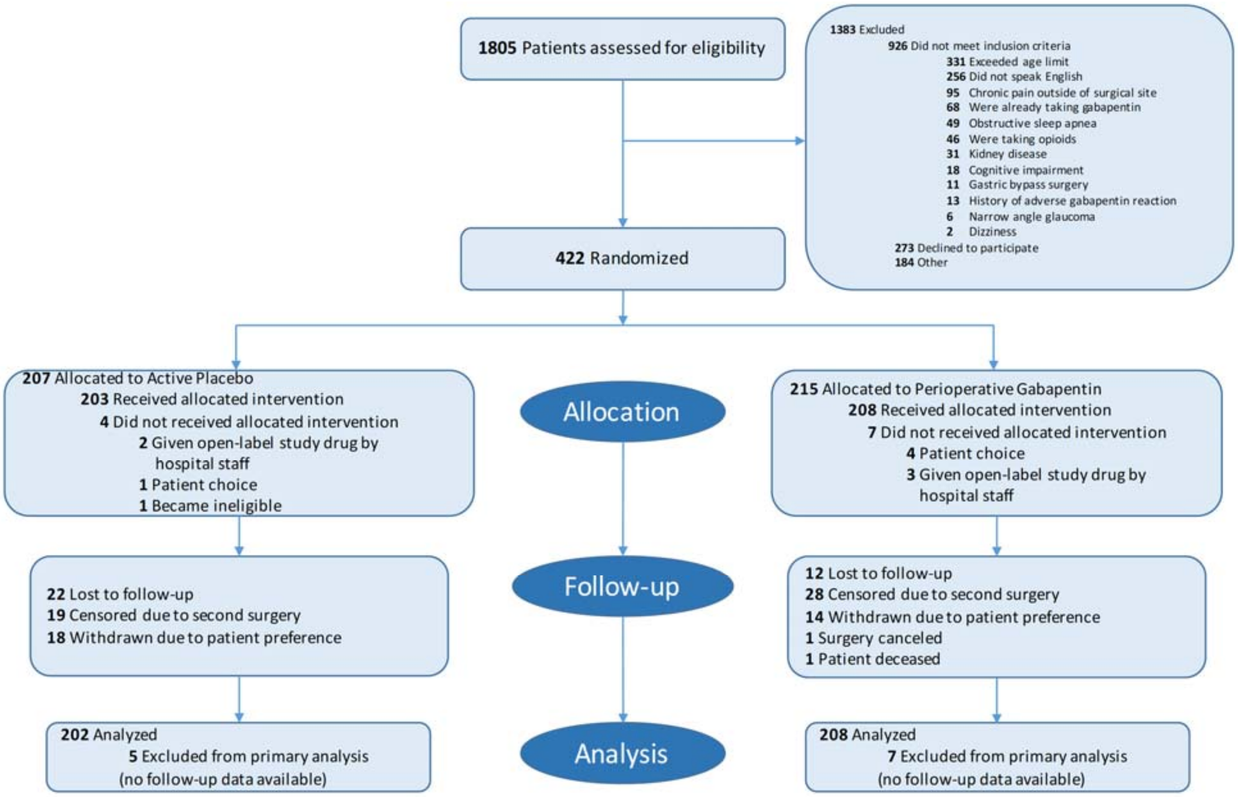

© 2019 Hah JM et al. *JAMA Network Open.*

eTable

High Pain Cluster of Average Pain Trajectories Preoperative Risk Factors[a]			
Characteristic	ARR	95% Confidence Interval	p-value
Female Sex	1.36	(1.08-1.70)	0.008
Baseline pain at surgical site (0-10)[b]	1.11	(1.07-1.15)	<0.001
History of Alcohol or Drug Abuse Treatment	1.90	(1.42-2.53)	<0.001
Received Active Placebo	1.27	(1.03-1.56)	0.03

[a]Modified Poisson Regression
[b]Every 1 point increase in the Numeric Rating Scale of Pain

© 2019 Hah JM et al. *JAMA Network Open.*
